# A morphometric study of posterior tibial slope differences by sex and ethnicity in a South African population

**DOI:** 10.1007/s00276-024-03551-2

**Published:** 2025-01-03

**Authors:** Erik Hohmann, Adri Nel, Reinette van Zyl, Natalie Keough, Nkhensani Mogale

**Affiliations:** 1 University of Pretoria, Pretoria, South Africa; 2 Burjeel Hospital for Advanced Surgery, Dubai, United Arab Emirates; 3 University of Warwick, Coventry, UK

**Keywords:** Posterior tibial slope, Morphology, anatomy, Sex, Race

## Abstract

**Purpose:**

Posterior tibial slope (PTS) influences knee kinetics and kinematics. The purpose of this study was to investigate morphology and variation within a sample of the black and white male and female population.

**Method:**

480 randomly selected lateral knee radiographs were included. The anterior tibial cortex angle (ATC), proximal anatomical tibial axis angle (PTAA) and the posterior tibial cortex angle (PTC) were measured using ImageJ 1.53e software. Between group differences (black male and females, white males and females) were analysed using one-way ANOVA.

**Results:**

Significant differences between the three different angles (*p* = 0.0001, F = 50.68) but no between group differences for the individual angle measurements (ATC, PTAA, PTC) between ethnicity and sex. For ATC, the angles between groups ranged from 14.20 + 2.81 degrees (Females Black), 14.62 + 3.6 degrees (Male Black), 15.18 + 3.68 degrees (Male White) to 15.54 + 3.21 degrees (Females White). For PTAA, the angles between groups ranged from 10.37 + 2.59 degrees (Females Black), 10.61 + 3.27 degrees (Male Black), 10.68 + 3.27 degrees (Male White) to 10.83 + 3.27 degrees (Females White). For PTC, the angles between groups ranged from 6.07 + 3.13 degrees (Females White), 6.13 + 3.7 degrees (Male White), 6.35 + 2.67 degrees (Females Black) to 6.62 + 3.16 degrees (Male Black).

**Conclusion:**

This study could not establish differences in posterior tibial slope angles between males and females and ethnicity. Significant differences between ATC, PTAA and PTC angles were observed and PTC angles were smaller when compared to PTAA and ATC angles. The ATC angles ranged between 13.18 and 16.57 degrees, the PTAA angles ranged 9.38–11.87 degrees and the PTC angles ranged between 5.03 and 7.62 degrees for all groups.

## Introduction

The posterior tibial slope (PTS) refers to the posterior inclination of tibial plateau in relation to a perpendicular line drawn over the longitudinal axis of the tibia [7, 30, 34]. Variations in PTS potentially influences kinetics, kinematics and biomechanics of the knee joint [22]. For example, an increased PTS decreases tibial sag and reduces load on the posterior cruciate ligament (PCL) whereas a decrease in PTS causes an anterior shift of the resting position of the tibia reducing load on the anterior cruciate ligament (ACL) [22]. An increased or decreased PTS also potentially alters the forces during dynamics tasks causing increased loads to the cruciate ligaments [23]. Increases in shear force and tibial translation can result in higher risk of injury to both the ACL and PCL [22]. Bernhardson et al. demonstrated that a decreased PTS was associated with a higher risk for PCL injuries [5]. The relationship between an increased PTS and ACL injuries has been demonstrated by various authors [13, 24, 25, 44].

Given these associations, slope changing osteotomies are now more frequently considered to not only reduce load on the ACL but also to reduce the risk of re-injury following surgical reconstruction [9, 28, 38]. In addition, it is also suggested to perform concurrent slope-reducing osteotomies in patients when the PTS is exceeding 12 degrees [38].

The tibial slope is also another important aspect to consider in knee replacement surgery [3, 43]. The biomechanical characteristics of knee replacement implants often require a specific inclination of the tibial plateau for knee kinematics, tibiofemoral contact area and long-term survival [41, 43].

At maturity, PTS ranges from 0 to 20 degrees and the absolute value depends on sex and the population group [22]. Frick reported a range between 4 and 7 degrees of PTS in the German White population [19]. Genin et al. measured PTS in a cadaver study using Caucasian specimens and showed that PTS varied between 0 and 18 degrees [20]. In the Chinese population PTS varied between 6 and 14 degrees [10] whereas the average PTS in the Japanese population was measured to be 11 degrees [33]. In the Saudi Arabian population, the mean PTS was 5.8 degrees in men and 6.6 degrees in women [2]. In the East African population, the mean PTS was measured to be 7 degrees [34].

For these reasons detailed knowledge on the tibial plateau morphology is critical and population groups with a larger PTS may not only be at higher risk for cruciate ligament injuries but may also require adjustment of the tibial plateau with knee replacement surgery. The purpose of this study was therefore to investigate the morphology and variation within a sample of the black and white male and female population. It was hypothesized that there would be significant differences between ethnic population group but no sex differences within an ethnic population group.

## Materials and methods

This study was designed as a retrospective cross-sectional study. The samples were obtained from a private radiological practice [Tesla Radiological Services, Pretoria, South Africa]. The sample consisted of 480 lateral knee radiographs which were randomly selected from the database by an independent research associate. The following inclusion criteria were applied: age between 18 and 80, true lateral knee radiograph as described by Lampignano et al. [i^31^], no radiographic evidence of previous surgery or fractures of the lower extremity and no radiographic evidence of degenerative changes. Radiographs were excluded if the images were under- or over rotated and if there was evidence of osteoarthritic changes. Ethical clearance was obtained from the ethics committee of the University of Pretoria, South Africa [ethical clearance: 360/2021].

### Posterior tibial slope measurements

The following angles were measured in this study: anterior tibial cortex angle (ATC), proximal anatomical tibial axis (PTAA) and posterior tibial cortex angle (PTC). The PTC angle was defined as the standard measure. Brazier et al. [7] demonstrated that this angle is not influenced by age, sex, height or body mass but angle values are typically smaller when compared to PTAA.

**Fig. 1:**
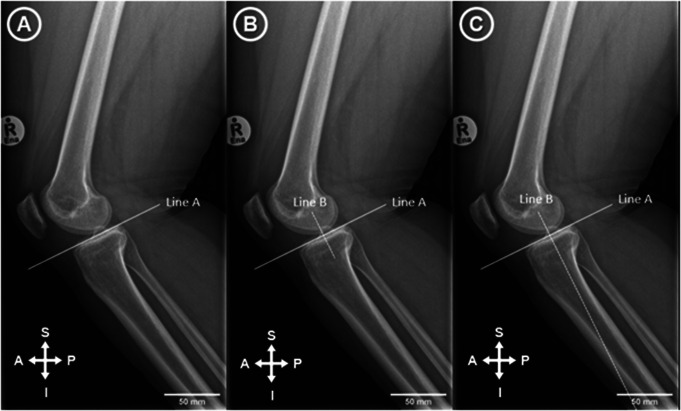
(**A**): An extended line was drawn to connect the most elevated anterior and posterior points of the tibial plateau (Line A). (**B**): A second perpendicular line was then drawn at the midpoint between the most elevated anterior and posterior points of the tibial plateau (Line B). (**C**): Line B was extended inferiorly to reach the bottom of the image

**Fig. 2:**
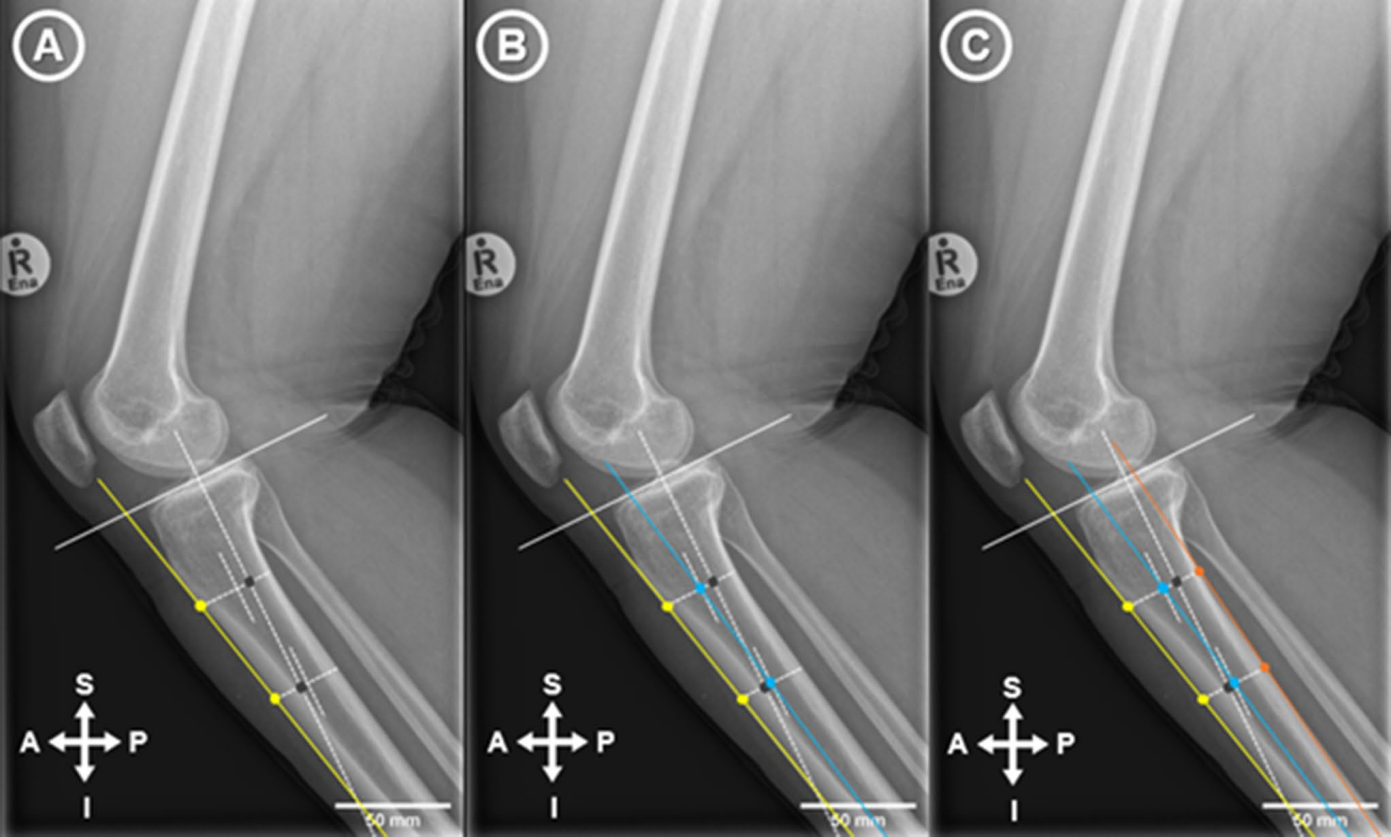
(**A**): The ATC angle was drawn by connecting the anterior points at 5 cm and 10 cm distal to the tibial plateau that extended past the tibial plateau line and distally to the most inferior aspect of the tibia visible in the image (yellow line). (**B**): The proximal tibial anatomical axis (PTAA) was drawn by connecting the midpoints of the horizontal lines drawn at 5 cm and 10 cm respectively and extending the line to the distal-most visible part of the tibia and past the tibial plateau line (blue line). (**C**): The posterior tibial cortex axis (PTC) was drawn by connecting the posterior points at 5 cm and 10 cm distal to the tibial plateau that was extended past the tibial plateau line (orange line) and distally to the most inferior aspect of the tibia visible in the image (Fig. 3.5 C)

Radiographs were imported into ImageJ 1.53e. An extended line was drawn to connect the most elevated anterior and posterior points of the tibial plateau (Tibial Plateau Line) (Fig. 1A). A second perpendicular line was then drawn from the midpoint between the most anterior and posterior point of the tibial plateau and extended inferior (Fig. 1B & C). The points on the second line that were 5 cm and10 cm distal to the tibial plateau were identified and perpendicular lines were drawn connecting the anterior and posterior cortex of the tibial shaft (Fig. 2A).

**Fig. 3:**
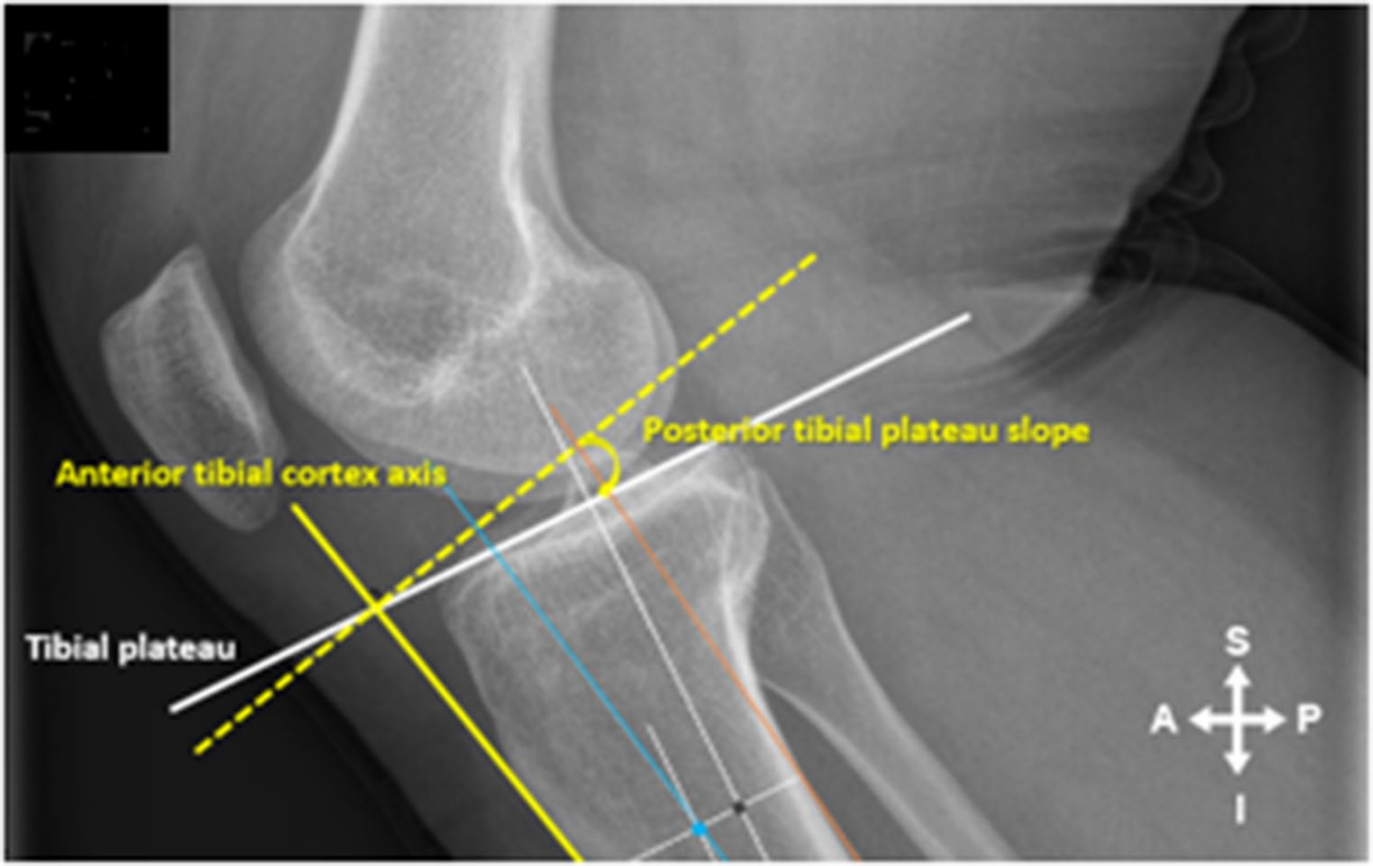
The angle between a line drawn 90 degrees to the ATC line and the Tibial Plateau line was then measured and represented the ATC angle

**Fig. 4:**
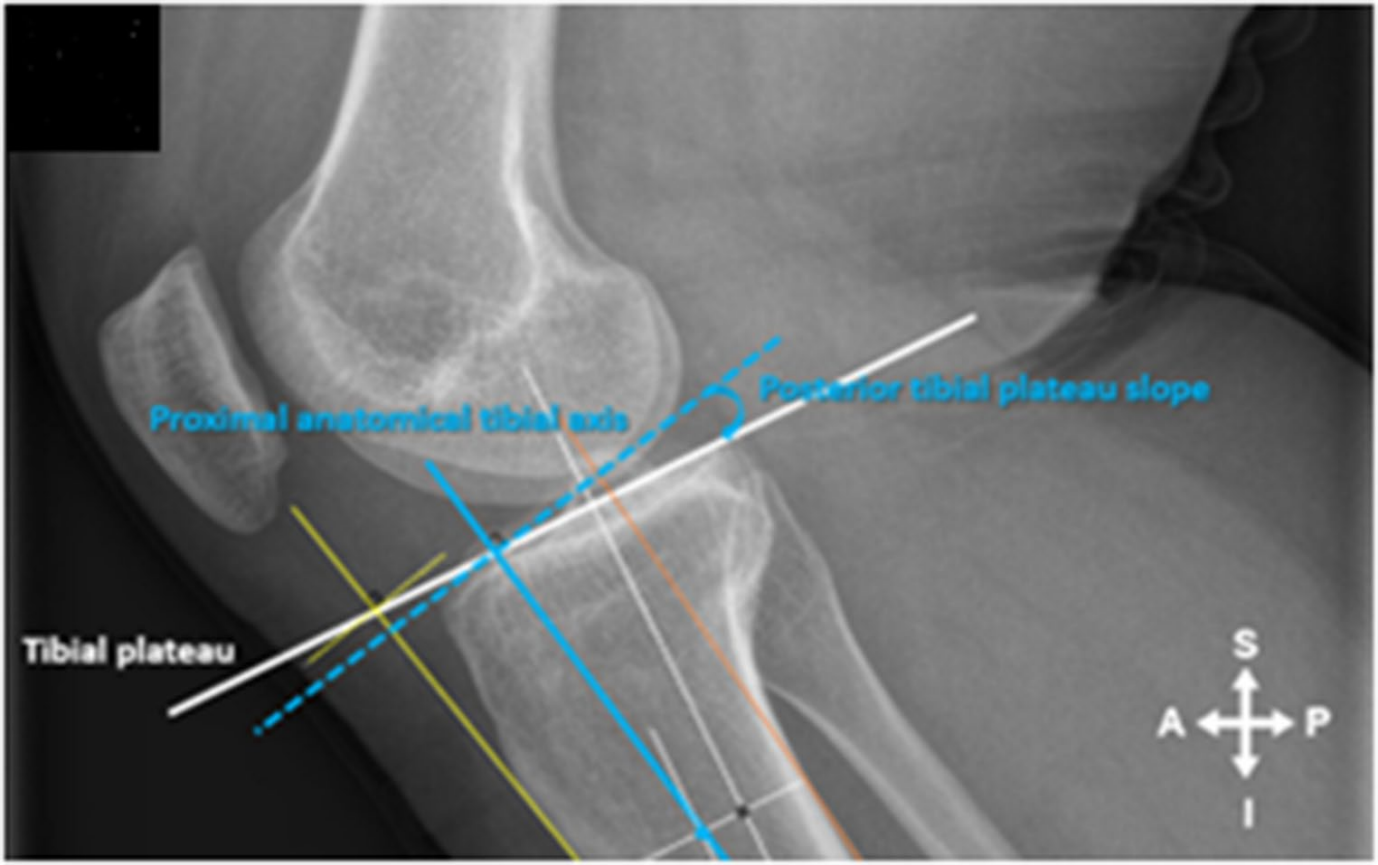
The angle between a line drawn 90 degrees to the PTAA line and the Tibial Plateau line was then measured and represented the PTAA angle

**Fig. 5:**
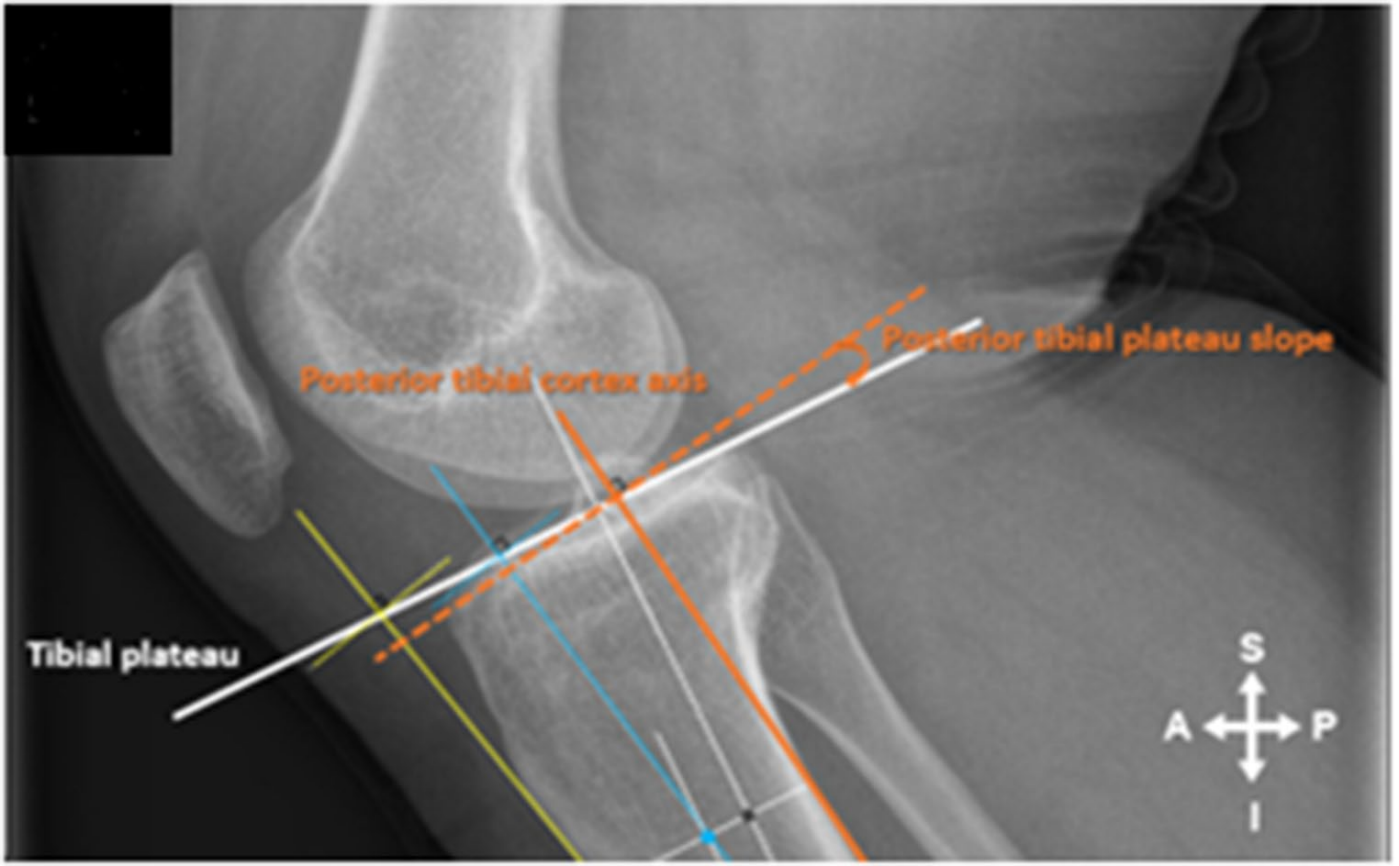
The angle between a line drawn 90 degrees to the PTC line and the Tibial Plateau line was then measured and represented the PTC angle

For the ATC angle the intersection of the connecting line (ATC yellow line) at 5 and 10 cm distal to the tibial plateau was connected and extended both proximal and distal (Fig. 2A). The angle between a line drawn 90 degrees to the ATC line and the Tibial Plateau line was then measured and represented the ATC angle (Fig. 3). For the PTAA angle the midpoint of the distal lines at 5 and 10 cm connecting the anterior and posterior cortex of the tibial shaft was established and a line was drawn between these two points extending both proximal and distal (PTAA blue line) (Fig. 2B). The angle between a line drawn 90 degrees to the PTAA line and the Tibial Plateau line was then measured and represented the PTAA angle (Fig. 4). For the PTC angle the intersection of the connecting line (PTC orange line) at 5 and 10 cm distal to the tibial plateau was connected and extended both proximal and distal (Fig. 2C). The angle between a line drawn 90 degrees to the PTC line and the Tibial Plateau line was then measured and represented the PTC angle (Fig. 5).

### Statistical analysis

Descriptive statistics were used for the distance and angle measurements. Mean length and angle, standard deviation, 95% confidence intervals and minimum and maximum values were calculated. Normal data distribution was assessed with the Shapiro-Wilks Test. Homogeneity of variance was verified with Levene’s test. Between group differences (black male and females, white males and females) were analysed using one-way ANOVA. If significant between group differences were observed Tukey’s simultaneous tests for differences of means was used. The relationships between PTC, ATC and PTAA were analysed with a linear regression model and ATC and PTAA were selected as the predictor variables.

Intra- and inter-rater reliability (ICC) were established by repeating the radiographic measures on two consecutive days on ten lateral knee radiographs by two independent research associates. The algorithm of Landis and Koch was used to assess the rate of agreement [32]. Values above 0.80 represented excellent agreement, values between 0.62 and 0.79 were considered good agreement, values between 0.41 and 0.61 indicated moderate agreement, and values below 0.4 suggested fair to poor agreement [32]. If the ICC data was considered to be above 0.8, all measures were performed by one researcher [xx].

An a-priori sample size calculation was performed using G*Power 3.1.9.2. The following variables were used: effect size 0.25, alpha 0.05, power 95%, four groups, three measures. Based on these variables a total sample size was required to achieve an actual power of 95.1%.

All analyses were conducted using STATA SE (Version 12.0; StataCorp, College Station, Texas, USA) for Windows.

## Results

Intra- and inter-rater reliability (ICC) was determined to be 0.97 for the intra-rater coefficient and 0.8 for the inter-rater coefficient. According to Landis & Koch [32] these values were excellent. All angle measurements were therefore performed by one examiner only [xx]. All data were normally distributed and Leven’s test revealed data homogeneity.

One-way ANOVA revealed that there were significant differences between the three different angles (*p* = 0.0001, F = 50.68). However, there were no significant between group differences for the individual angle measurements (ATC, PTAA, PTC), ethnicity and sex differences were not significant. For ATC, the angles between groups ranged from 14.20 + 2.81 degrees (Females Black), 14.62 + 3.60 degrees (Male Black), 15.18 + 3.68 degrees (Male White) to 15.54 + 3.21 degrees (Females White) (Table 1). For PTAA, the angles between groups ranged from 10.37 + 2.59 degrees (Females Black), 10.61 + 3.27 degrees (Male Black), 10.68 + 3.27 degrees (Male White) to 10.83 + 2.87 degrees (Females White) (Table 1). For PTC, the angles between groups ranged from 6.07 + 3.13 degrees (Females White), 6.13 + 3.70 degrees (Male White), 6.35 + 2.67 degrees (Females Black) to 6.62 + 3.16 degrees (Male Black) (Table 1).

**Table 1:** Posterior tibial slope angles for sex and ethnicity

	Group	Mean	STDEV	Minimum	Maximum	95% Confidence Interval
ATC	Female Black	14.20	2.81	8.46	22.30	13.18–15.14
	Female White	15.54	3.21	5.39	22.62	14.50-16.57
	Male Black	14.62	3.60	5.86	22.70	13.28–15.62
	Male White	15.18	3.68	4.72	22.14	14.14–16.21
PTAA	Female Black	10.37	2.59	5.31	17.09	9.38–11.35
	Female White	10.83	2.87	2.76	17.88	9.80-11.87
	Male Black	10.61	3.27	2.16	19.61	9.62–11.61
	Male White	10.68	3.27	0.80	16.91	9.64–11.71
PTC	Female Black	6.35	2.67	0.56	12.03	5.36–7.33
	Female White	6.07	3.13	0.17	13.88	5.03–7.10
	Male Black	6.62	3.16	0.79	16.91	5.62–7.62
	Male White	6.13	3.70	2.78	12.06	5.10–7.16

The linear regression model revealed that both ATC and PTAA were predictors of the PTC angle. The adjusted R-Square was 0.893 and significant (*p* = 0.0001). Tukey’s simultaneous 95% confidence intervals showed no overlap between the pairs (PTAA-ATC, PTC-ATC, PTC-PTAA) suggesting that the corresponding means are significantly different.

**Fig. 6:**
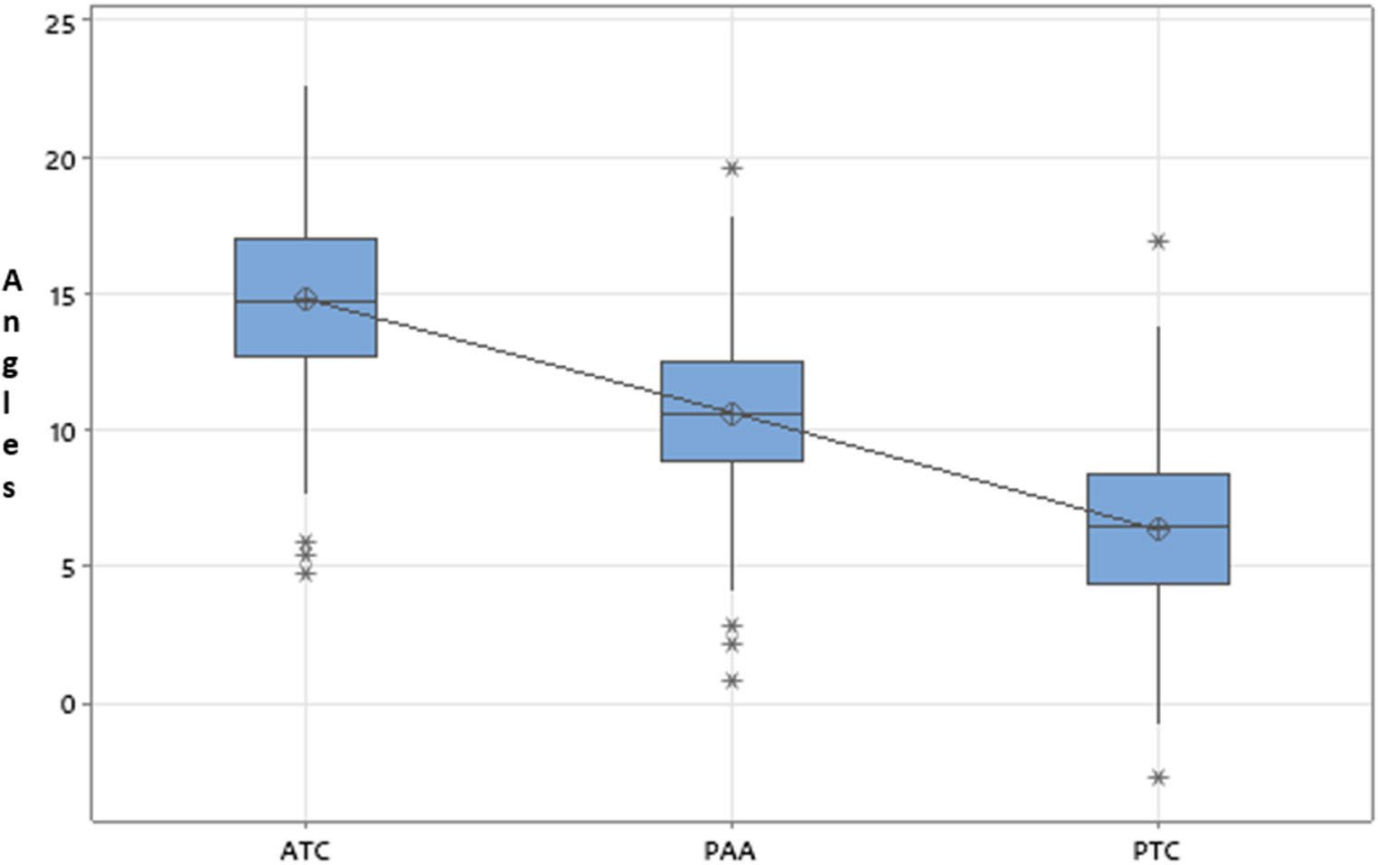
The linear regression model revealed that both ATC and PTAA were predictors of the PTC angle and the regression equation demonstrated a linear relationship

The regression equation demonstrated a linear relationship (Fig. 6):

$${\rm{PTC = - 0}}{\rm{.879 - 06437 ATC + 1}}{\rm{.5749 PTAA}}$$

## Discussion

The results of this study could not establish differences in posterior tibial slope angles between males and females and ethnicity. However, significant differences between ATC, PTAA and PTC angles were observed. PTC angles were smaller when compared to PTAA and ATC angles. The 95% confidence intervals demonstrated no overlap between the three angles. The ATC angles ranged between 13.18 and 16.57 degrees, the PTAA angles ranged 9.38–11.87 degrees and the PTC angles ranged between 5.03 and 7.62 degrees for all groups. The regression analysis also revealed that both ATC and PTAA were predictors of the PTC. This relationship was linear.

The PTC angles were smaller than the PTAA angles and the observed differences are within the limits outlined by Brazier et al. [7]. ICC data showed excellent agreement and one could therefore safely assume that the results are reliable and valid.

Muthuuri has previously reported on the PTS in an East African sample of 79 non-arthritic patients using the PTAA angles [34]. The authors established a mean angle of 6.7 degrees with a 95% confidence interval ranging from 6.0 to 7.4 degrees [34]. The results are not comparable and the PTS in the South African Sample is substantially larger. These differences could potentially be explained by the origin of the East and South African population. Paleontological and genetic evidence suggests that the East African population Nilo-Saharan Cushitic group with a substantial intermix of Ottomans, Arabian and Semitic groups whereas the black Southern African population most likely originates from the Niger-Kordofanian group [8]. This explanation is supported by several studies [2, 15, 29, 34].

Aljuhani et al. reported PTS in a Saudi Arabian sample of 285 patients using MRI [2]. They showed that the PTS angles were 6.12 + 3.14 degrees which were quite similar to the slope angles reported by Muthuuri [34]. In a similar study Kacmaz et al. investigated PTS in a Turkish population and determined a mean slope of 8.36 degrees [29]. Didia & Jaja investigated PTS in a Nigerian population and reported a mean slope 12.3 degrees which is similar to the results of our study [15].

Ethnicity can affect PTS, however, the current evidence is conflicting. De Boer et al. showed differences between black and white subjects [14]. Clinger et al. demonstrated that African-Americans and Asian-Americans have an increased PTS in comparison with whites [12]. Bisicchia et al. compared blacks and whites and indicated that whites have a smaller PTS when compared to whites [6]. However, the authors Bisicchia et al. suggested that despite ethnic differences, the observed differences might be too small to be clinically relevant [6]. The results by de Boer et al. [14] showed a difference of 2.8 degrees with larger slopes in the black population while the results by Clinger et al. [12] indicated a difference of 1.7 degrees with a larger slope in African- Americans and Asian-Americans and Bisicchia et al. [6] reported a 2.2 degree difference with large slopes documented in the black population. In contrast, Haddad et al. [21] could not find any differences between the ethnic groups. The results of this study support the findings by Haddad et al. [21]. We could not find any differences between ethnic groups and the mean slope in both black and white subjects was within 0.55 degrees.

Similar to ethnicity, sex can affect tibial slope but the current evidence is also conflicting. Haddad et al. found significant differences between the sexes but these differences were rather small [21]. The PTS in females was 0.8 degrees larger and despite statistical significance, the clinical relevance is questionable [21]. Bisicchia et al. showed no between sex differences in the white population but reported a significant mean difference of 1.2 degrees with a larger slope in females [6]. Similar to Hadad et al. these differences are unlikely to be clinically relevant [21]. Clinger et al. did not find any sex-based differences [12]. The results of this study could also not find any differences between sex and the mean slope in both the male and female cohort was within 0.45 degrees.

It is widely acknowledged that PTS influences knee kinetics and kinematics [23]. In anterior cruciate ligament deficient and reconstructed knees, knee functionality has a direct and linear relationship with tibial slope [23]. Tibial slope also influences the length-tension relationships and musculotendinous stiffness of the supporting muscle groups and has an indirect effect on knee stability [22, 23]. PTS has also been associated with an increased risk of cruciate ligament injuries [4, 13, 23–25, 40, 44]. Dracic et al. [16] recently evaluated the cutoff value for the posterior tibial slope (PTS) in anterior cruciate ligament (ACL) injuries. Their findings suggest that a PTS exceeding 10 degrees is associated with an 11-fold increased risk of ACL graft failure. Based on these results, they recommend considering a slope-reducing osteotomy in patients with a high PTS to mitigate this risk. These values are slightly lower than those previously reported, where slope-reduction surgery was recommended for cases in which the posterior tibial slope exceeded 12–13 degrees [38, 39, 42]. Currently, the anterior closing wedge osteotomy is the most widely used surgical technique for posterior tibial slope reduction, typically aiming for a correction of 10 degrees [28, 42].

With knee replacement surgery it is suggested not to cut a slope of greater than 8 degrees as this may have an effect on knee stability, function and survival of the knee implant [14].

In general, recommendations for PTS values in knee replacement surgery are implant-specific and range from 0 to 10 degrees [4, 37]. For posterior cruciate-retaining knee prostheses, slopes of 3–7 degrees are typically recommended, while for posterior-stabilizing implants, values of less than 5 degrees are suggested. Robotic-assisted knee replacement surgery aims to position implants in an anatomo-functional position to allow native knee alignment and a natural functional pattern [36]. In this context, the posterior tibial slope should ideally be set to up to 3 degrees for cruciate-retaining (CR) implants and 0 degrees for posterior-stabilizing implants [37]. In contrast, a higher posterior tibial slope (PTS) may have beneficial effects on patellofemoral contact force and influence quadriceps force. Okamoto et al. [35] used a musculoskeletal model to demonstrate that while patellofemoral force decreased with an increasing slope, it also led to a reduction in maximal quadriceps force. The authors suggested aiming for a slope of less than 5 degrees to balance these opposing forces.

An increase in tibial slope also seems to increase the risk of meniscus injuries [1] which is possibly related to the meniscus as a slope reducing structure that acts as a chock block in patients with an increased PTS [26, 27].

Given these considerations, it is important to obtain PTS measures of the normal and healthy population [10]. In current clinical practice, little emphasis is placed on the exact measurement of PTS and remains the “unknown size” of the knee joint [17, 18].

### Limitations

This study had several limitations. PTS was measured on conventional radiographs which could have introduced measurement error. South Africa has multiple ethnic groups and it is possible that one or more of these ethnic groups were over- or under-represented in the study cohort. The proximal tibia is a three-dimensional structure, 2D lateral radiographs may not accurately reflect the native 3D tibial slope. In contrast to long-leg radiographs, short lateral knee radiographs may over- or underestimate the true slope and Faschingbauer et al. [18] have shown that short radiographs overestimate the slope by an average of 3 degrees.

## Conclusions

The results of this study could not establish differences in posterior tibial slope angles between males and females and ethnicity. Significant differences between ATC, PTAA and PTC angles were observed and PTC angles were smaller when compared to PTAA and ATC angles. The ATC angles ranged between 13.18 and 16.57 degrees, the PTAA angles ranged 9.38–11.87 degrees and the PTC angles ranged between 5.03 and 7.62 degrees for all groups. The regression analysis revealed a linear relationship and showed that both ATC and PTAA were predictors of the PTC.

## Data Availability

No datasets were generated or analysed during the current study.
