# Correction: A morphometric study of posterior tibial slope differences by sex and ethnicity in a South African population

**DOI:** 10.1007/s00276-025-03639-3

**Published:** 2025-04-28

**Authors:** Erik Hohmann, Adri Nel, Reinette van Zyl, Natalie Keough, Nkhensani Mogale

**Affiliations:** 1 University of Pretoria, Pretoria, South Africa; 2 Burjeel Hospital for Advanced Surgery, Dubai, United Arab Emirates; 3 University of Warwick, Coventry, UK

**Correction: Surgical and Radiologic Anatomy (2024) 47:52**

10.1007/s00276-024-03551-2

In this article the author name Natalie Keough was incorrectly written as Natalie Natalie.

The original article has been corrected.

